# Research Progress of Rare-Earth-Functionalized Carbon Electrodes for Vanadium Redox Flow Batteries

**DOI:** 10.3390/ma19173723

**Published:** 2026-09-01

**Authors:** Jingya Li, Chen Chen, Huimin Ma, Feng Wang, Yu Cheng, Ruihua Guo

**Affiliations:** 1 Baotou Research Institute of Rare Earths, Baotou 014030, China; 2 School of Materials Science and Engineering, Inner Mongolia University of Science & Technology, Baotou 014010, China

**Keywords:** vanadium flow battery, rare-earth functional materials, carbon-based electrodes, electrocatalysts, interface regulation

## Abstract

**Highlights:**

Rare-earth modification is divided into surface loading and bulk doping for VRFB carbon electrodes. The electronic–geometric synergistic catalysis mechanism toward vanadium redox reactions is clarified. Industrial bottlenecks and full-lifecycle recycling strategies of rare-earth electrodes are prospected.

**What are the main findings?**

- Two rare-earth modification strategies (surface loading and bulk doping) for VRFB carbon electrodes are systematically classified, revealing their different catalytic behaviors.
- The electronic–geometric synergistic mechanism is summarized to explain the promotion effect of rare earths on vanadium redox kinetics.

**What are the implications of the main findings?**

- The classification of surface loading and bulk doping provides targeted guidance for the rational design of rare-earth-modified carbon electrodes for vanadium flow batteries.
- The electronic–geometric synergistic mechanism offers a universal theoretical framework to develop high-efficiency rare-earth electrocatalysts toward vanadium redox reactions.

**Abstract:**

Commercial carbon-based electrodes such as graphite felt and carbon felt in all-vanadium redox flow batteries suffer from inherent drawbacks, including slow vanadium ion redox kinetics, insufficient intrinsic catalytic activity, fiber corrosion, and functional group loss under strong acidic oxidative conditions, significantly limiting battery energy efficiency and long-term operational reliability. Rare-earth elements, with their unique 4f electron shell structure, tunable electronic levels, abundant surface oxygen vacancy defects, and strong coordination ability, offer a dual pathway—electronic and microstructural modulation—to optimize the interfacial electrocatalytic behavior of carbon electrodes, providing a novel materials system to overcome electrode performance bottlenecks in vanadium batteries. This review systematically summarizes recent advances in rare-earth-functionalized carbon electrodes and electrocatalysts for vanadium redox flow batteries, elaborating on core modification strategies, performance enhancement trends, and synergistic catalytic mechanisms. It also presents quantitative experimental results from the literature to clearly demonstrate the benefits: CeO_2_-modified graphite felt at 0.2 wt% shows a 10.8% increase in energy efficiency compared to pristine graphite felt at a current density of 200 mA·cm^−2^, while multi-rare-earth co-doped carbon electrodes achieve a 65% reduction in charge transfer resistance relative to unmodified electrodes. The review systematically categorizes two dominant modification routes—surface nano-decoration with rare-earth oxides and lattice bulk doping with rare-earth elements—and summarizes design principles and enhancement mechanisms of diverse composite catalytic systems, including rare-earth–carbon nanocomposites, rare-earth-based heterojunctions, and porous rare-earth catalysts. It further analyzes critical challenges in current research, such as unclear long-term stability mechanisms, high costs of high-purity rare-earth raw materials, immature large-scale fabrication processes, and limited in situ dynamic characterization techniques. Compared with existing reviews, this work clearly distinguishes between surface loading and lattice doping as two distinct rare-earth modification approaches, clarifying their differences in active site formation, electronic regulation logic, and cycling stability. It establishes a comprehensive theoretical framework for the coupled electronic–geometric effects in rare-earth-modified carbon electrodes, linking the intrinsic physicochemical properties of rare earths, material microstructure design, and battery electrochemical performance. Moreover, it innovatively proposes a pathway toward full-lifecycle recycling and reuse of rare-earth-based catalytic electrodes for industrial implementation. This review provides a complete theoretical foundation for developing high-performance, long-cycle, low-cost vanadium redox flow battery electrode materials and supports their engineering scale-up, contributing to the development of large-scale, long-duration energy storage technologies.

## 1. Introduction

Driven by the ‘Dual Carbon’ strategic objectives, the global energy structure is undergoing an accelerated transition towards low-carbon sources. The large-scale integration of renewable energy sources such as wind and solar power poses significant challenges due to their inherent volatility and intermittency, creating an urgent need for large-scale, long-duration energy storage technologies to ensure the safe and stable operation of the power grid [1,2]. VRFBs, owing to their unique advantages—including independent design of power and capacity, long cycle life (>20,000 cycles), rapid response, intrinsic safety and environmental friendliness [3,4]—have demonstrated irreplaceable application value in areas such as grid peak shaving and energy storage to support renewable energy. They are regarded as one of the most promising large-scale energy storage technologies [5,6].

As the core site of electrochemical reactions in VRFBs, the material properties of the electrodes directly determine the battery’s energy conversion efficiency, power density and long-term operational reliability. Currently, commercial VRFBs primarily utilize porous carbon materials such as graphite felt and carbon felt as electrodes. These materials, as compared in Table 1, possess the advantages of low cost, good electrical conductivity, and high specific surface area, but suffer from significant drawbacks like insufficient catalytic activity, poor stability, functional group loss, and fiber corrosion under acidic oxidative conditions [2]. Although these materials possess good electrical conductivity and chemical stability, their intrinsic electrocatalytic activity is insufficient, and their ability to catalyze vanadium ion redox reactions is limited, making this a key bottleneck constraining further improvements in battery performance [7]. The cathode reaction involves oxygen atom transfer and the restructuring of the V=O bond; the reaction pathway is complex, the kinetics are extremely slow, and significant polarization losses are prone to occur. The anode reaction kinetics are relatively favorable; however, at high current densities, polarization still occurs due to a shortage of active sites [8,9,10]. Furthermore, under strongly acidic and highly oxidative conditions, carbon electrodes are also subject to issues such as the loss of surface functional groups and the corrosion and degradation of carbon fibers, leading to irreversible deterioration in battery performance [11].

To overcome the performance bottlenecks of carbon-based electrodes, researchers have developed various strategies, including surface functional group modification, heteroatom doping and nanomaterial modification [12]. Rare-earth elements (scandium, yttrium and lanthanide elements), as summarized in Table 2, owing to their unique 4f electron shell structure, exhibit tunable electronic energy levels, abundant surface defects and reversible redox properties, which are highly suited to the core catalytic requirements of VRFB electrodes [13,14,15]. These properties enable rare earths to act as ‘electron shuttles’ to establish efficient charge transfer pathways and activate vanadium oxygen ions [16]. Consequently, rare-earth-functionalized materials offer a new research direction for overcoming the performance bottlenecks of carbon-based electrodes, and a series of advances have already been achieved.

**Table 1:** Comprehensive comparison of physicochemical properties, advantages, disadvantages, and typical applications of flow battery electrode materials.

Materials	Advantages	Disadvantages	Key Physicochemical Properties	Typical Application/Reference
Carbon felt/Graphite felt	Low cost (~$5–15/m^2^), good electrical conductivity (~10^2^–10^3^ S·m^−1^), high specific surface area (~0.3–1.0 m^2^·g^−1^), good chemical stability in 2–5 M H_2_SO_4_; 3D fiber network facilitates electrolyte permeation [2]	Insufficient intrinsic electrochemical activity for VO^2+^/VO_2_^+^; poor long-term stability under high current density; surface oxygen-containing groups gradually lost during cycling; fiber corrosion at >1.7 V vs. SHE [1,2]	SSA~0.3–1.0 m^2^·g^−1^; σ~10^2^–10^3^ S·m^−1^; 3D fiber network; pore size 10–50 μm	Most widely used commercial VRFB electrode; benchmark material in academic research [1,2]
Carbon paper	High specific surface area (~0.5–1.5 m^2^·g^−1^), good chemical stability, uniform pore structure, high mechanical strength; widely used in PEMFC and VRFB [5,6]	Poor hydrophilicity (water contact angle >120° without treatment); insufficient electrochemical activity; relatively high cost; brittle and difficult to handle [2,3,4,5,6,7]	SSA~0.5–1.5 m^2^·g^−1^; σ~10^3^ S·m^−1^; uniform pore~5–20 μm; high tensile strength	PEMFC, VRFB, zinc–bromine flow batteries; lab-scale high-performance cells [5,6]
Carbon fiber fabric	Excellent energy efficiency under optimized conditions; good flexibility and mechanical strength; woven structure provides ordered electrolyte flow channels [7]	Low specific surface area (<0.3 m^2^·g^−1^); prone to oxidative degradation during charge–discharge cycling; limited active sites on smooth fiber surface [11]	SSA < 0.3 m^2^·g^−1^; σ~10^3^ S·m^−1^; woven plain/twill structure; fiber diam. 7–10 μm	Flexible batteries, wearable electronics, small-scale VRFB prototypes [7]
Graphite	Excellent electrical conductivity (~10^4^ S·m^−1^), superior chemical inertness, low cost in bulk form; flat surface [15]	Extremely low specific surface area (<0.01 m^2^·g^−1^); prone to degradation at high anodic potentials; poor mass transport due to non-porous structure [15]	SSA < 0.01 m^2^·g^−1^; σ~10^4^ S·m^−1^; non-porous dense structure; high crystallinity	Electrochemical analysis, reference electrode substrates, basic mechanism studies [15]
Carbon cloth	High electrical conductivity, excellent flexibility, good mechanical strength, woven structure with interconnected pores; suitable for flow-through electrode design [8,9]	Relatively low specific surface area; hydrophobic surface requires activation treatment; higher cost than carbon felt [8,9]	SSA~0.5–2.0 m^2^·g^−1^; σ~10^3^–10^4^ S·m^−1^; woven structure; good flexibility	Flow-through electrode design, flexible VRFB, zinc–air batteries [8,9]
Graphene/CNT composite paper	Ultra-high specific surface area (>100 m^2^·g^−1^ for graphene), exceptional electrical conductivity (~10^4^–10^5^ S·m^−1^), abundant edge defects as active sites [10,12]	High manufacturing cost; tendency to agglomerate and restack; challenging to achieve uniform dispersion in porous electrodes; scalability remains limited [10,12]	SSA > 100 m^2^·g^−1^; σ~10^4^–10^5^ S·m^−1^; nanoscale pores; high defect density	High-power-density VRFB, supercapacitors, advanced composite electrodes [10,12]
Carbon aerogel	Ultra-high specific surface area (400–1000 m^2^·g^−1^), hierarchical porous structure, tunable pore size distribution, low density [13,14]	Poor mechanical strength and brittleness; high preparation cost; complex synthesis process (supercritical drying); limited commercial availability [13,14]	SSA 400–1000 m^2^·g^−1^; σ~10^2^ S·m^−1^; hierarchical micro/meso/macropores; ultra-low density	High-energy-density storage, electrochemical capacitors, catalyst supports [13,14]

**Table 2:** Typical rare-earth catalytic elements: valence states, representative compounds, catalytic mechanisms, and applicable electrodes.

Elements	Main Mechanism of Action	Suitable Electrodes	Typical Valence/Representative Compound	Key Catalytic Feature
Ce	Abundant reversible oxygen vacancies (Ce^3+^/Ce^4+^ redox couple) promote charge transfer; acts as ‘electron shuttle’ to accelerate VO^2+^/VO_2_^+^ kinetics; CeO_2_ is the most studied rare-earth oxide [17]	Positive terminal (VO^2+^/VO_2_^+^) is particularly prominent; also effective at negative terminal under optimized loading [17,18]	Ce^3+^/Ce^4+^; CeO_2_ (fluorite structure)	Most mature and widely studied; excellent oxygen vacancy reversibility; high natural abundance
Pr/Tb	Similar multivalent catalytic activity to Ce (Pr^3+^/Pr^4+^, Tb^3+^/Tb^4+^); enables efficient electron transfer at both anode and cathode; oxygen vacancy formation energy lower than CeO_2_ [19]	Both positive and negative terminals; bifunctional catalytic capability [19]	Pr^3+^/Pr^4+^; Tb^3+^/Tb^4+^; Pr_6_O_11_, Tb_4_O_7_	Bifunctional catalysis at both electrodes; lower oxygen vacancy formation energy than CeO_2_
Nd	Nd^3+^ binds to organic ligands or carbon surface functional groups, reducing charge transfer impedance; modulates electronic structure of carbon matrix; enhances V^2+^/V^3+^ redox kinetics [20]	Both positive and negative terminals; particularly effective in composite/co-doped systems [20,21,22,23]	Nd^3+^; Nd_2_O_3_, Nd–organic complexes	Strong coordination with organic ligands; effective impedance reduction
Gd/Sm	Doping into CeO_2_ lattice increases oxygen vacancy concentration via charge compensation (Gd^3+^/Sm^3+^ replacing Ce^4+^); enhances ionic conductivity and catalytic activity [24]	Both positive and negative terminals; commonly used as dopants in CeO_2_-based solid solutions [24]	Gd^3+^; Sm^3+^; Gd_2_O_3_, Sm_2_O_3_, Ce_1-x_Gd_x_O_2−δ_	Charge-compensation doping dramatically increases oxygen vacancy density
La	La^3+^ with stable +3 valence introduces lattice distortion and topological defects in carbon matrix; enhances oxygen-containing functional group anchoring; improves wettability and active site density [21,25]	Both terminals; effective in bulk doping and composite catalyst supports [21,25]	La^3+^; La_2_O_3_, La(OH)_3_	Lattice distortion and defect engineering; enhances wettability
Y	Y^3+^ small ionic radius enables deep lattice penetration; stabilizes oxygen vacancies via strong Y–O bonds; promotes electron transfer at carbon–electrolyte interface [22]	Both terminals; suitable for high-temperature doping and carbon nanofiber composites [22]	Y^3+^; Y_2_O_3_, Y-doped carbon	Small ionic radius for deep doping; strong Y–O bond stabilizes vacancies
Eu/Dy	Eu^2+^/Eu^3+^ and Dy^3+^ redox pairs provide additional electron transfer pathways; strong spin–orbit coupling modulates d-band centre of adjacent carbon atoms; enhances bifunctional catalysis [26]	Both terminals; promising for co-doping systems with Ce or Pr [26]	Eu^2+^/Eu^3+^; Dy^3+^; Eu_2_O_3_, Dy_2_O_3_	Strong spin–orbit coupling; additional redox pairs for electron shuttling
Yb/Er	Yb^2+^/Yb^3+^ and Er^3+^ with high coordination numbers form stable complexes with vanadium ions; facilitate proton-coupled electron transfer; improve reaction reversibility [23]	Positive terminal primarily; under investigation for negative terminal catalysis [23]	Yb^2+^/Yb^3+^; Er^3+^; Yb_2_O_3_, Er_2_O_3_	High coordination number; facilitates proton-coupled electron transfer

Currently, research into rare-earth-modified VRFB electrodes has largely focused on validating the performance of individual modification strategies, with a lack of systematic analysis of the relationships between intrinsic properties of rare earths, material structural design, electrode performance enhancement and catalytic mechanisms. Therefore, this review systematically categorizes rare-earth modification into surface modification (Section 3.1) and bulk doping (Section 3.2) and analyzes progress in composite catalyst design (Section 4). Accordingly, this paper provides a systematic review of progress in the application of rare-earth-functionalized materials in the field of VRFB electrodes and electrocatalysts. It offers an in-depth analysis of the core strategies for rare-earth modification, the patterns of performance enhancement, and the mechanisms of electronic–geometric synergy; identifies the key challenges facing current research; and outlines future research directions, including lifecycle recycling strategies, with the aim of providing a theoretical framework for the development of high-performance, long-life, and low-cost VRFB electrode materials.

## 2. Kinetics Bottlenecks and Key Requirements for Vanadium Redox Flow Battery Electrode Reactions

### 2.1. The Nature of Electrode Reaction Kinetics and Key Bottlenecks

The electrode reactions in VRFBs are complex solid–liquid interface processes involving multi-electron transfer and the coordinated participation of protons [27]. At the negative pole, a VO^2+^/VO_2_^+^ redox reaction takes place:

VO^2+^ + H_2_O → VO_2_^+^ + 2H^+^ + e^−^ (1)

This reaction is the rate-limiting step in the entire cell; its core bottleneck lies in the fact that it involves oxygen atom transfer and the breaking and reformation of the V=O bond. On conventional carbon-based electrodes, the kinetic process is extremely slow and the activation energy is high, making it the primary source of polarization loss in the cell [28]. At the positive pole, the following redox reaction takes place:

V^3+^ + e^−^ → V^2+^ (2)

**Figure 1:**
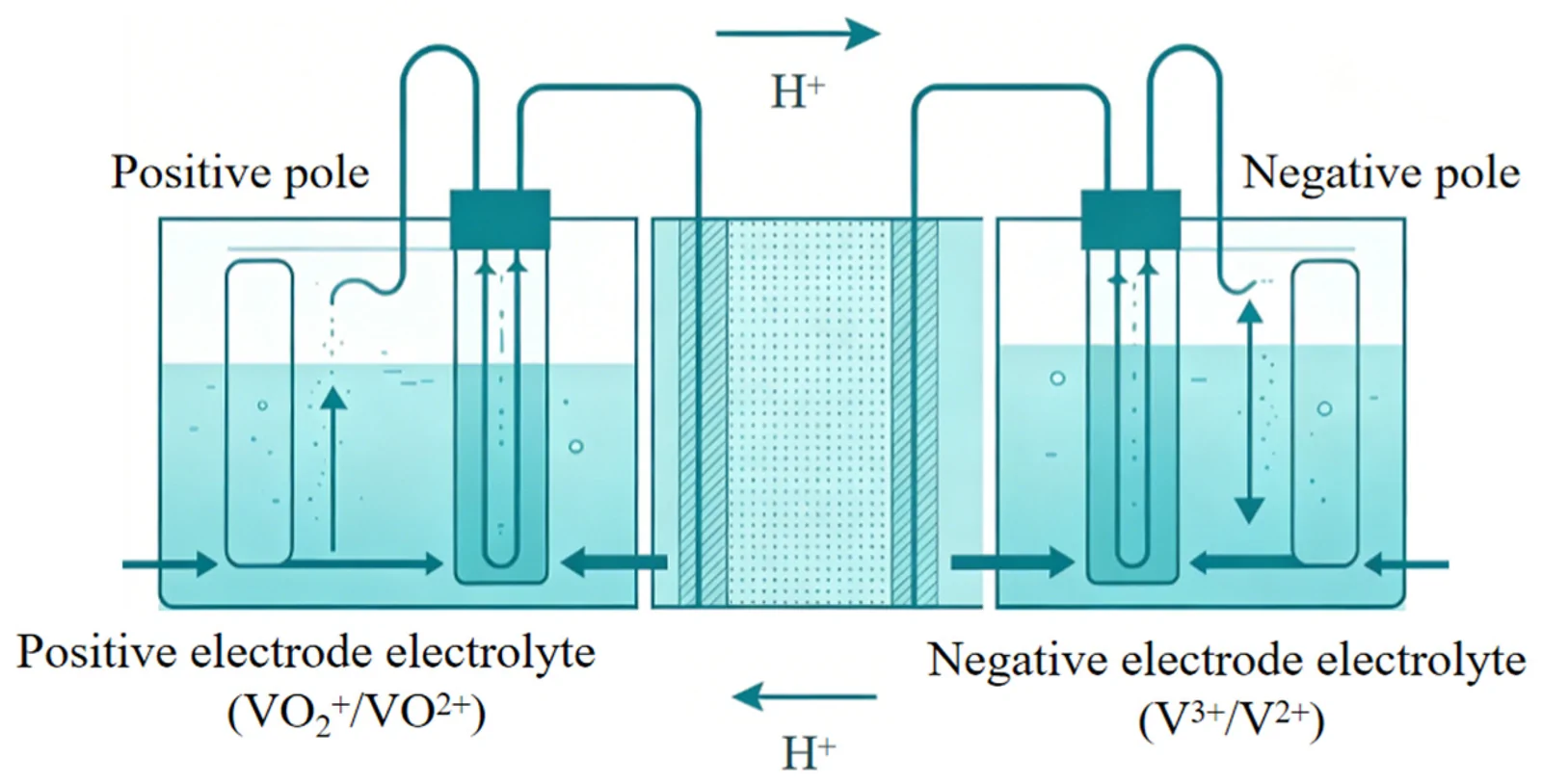
Schematic diagram of vanadium redox flow battery operation. The partial graphical elements were produced by AI image-generation tools. The authors have modified, reviewed and validated all scientific information contained in this figure to guarantee scientific accuracy.

Although the reaction involves only single-electron transfer, resulting in relatively favorable kinetics, concentration polarization and charge transfer impedance still occur at high current densities due to a shortage of active sites on the carbon material surface. In addition to slow kinetics, carbon-based electrodes also suffer from stability issues during long-term cycling, such as the loss of oxygen-containing functional groups, leading to a decline in battery performance (see Figure 1) [29].

A comprehensive comparison of the advantages and disadvantages of different flow battery electrode materials is presented in Table 1. Carbon felt/graphite felt, while low cost and conductive, have insufficient activity and poor stability. Other materials like carbon paper, carbon fiber fabric, and graphite also suffer from limitations in hydrophilicity, activity, or degradation [1,2,5,6,7,11,15]. Rare-earth modification is introduced precisely to address these intrinsic limitations.

### 2.2. Core Requirements for an Ideal Electrocatalyst in Vanadium Flow Batteries

Taking into account the electrode reaction characteristics and actual operating conditions of vanadium flow batteries, an ideal electrode electrocatalyst must simultaneously meet three core requirements: high catalytic activity, high electrical and mass transfer efficiency, and strong operational stability [17,30,31], Rare-earth elements, owing to their intrinsic physicochemical properties—such as electronic structures, abundant surface defects and strong coordination capabilities—can precisely enhance catalytic activity, create high-density active sites and achieve stable bonding with carbon matrices. As they are highly suited to the aforementioned requirements, as summarized in Table 2, they represent ideal functional materials for the modification of electrodes in vanadium flow batteries.

## 3. Core Strategies and Performance Enhancement of Rare-Earth-Modified Carbon-Based Electrodes

Currently, rare-earth-functionalized carbon-based electrodes have primarily evolved along two mainstream technical pathways: surface nanomodification and bulk element doping. The former focuses on creating high-density active sites on the surface of carbon materials, whilst the latter concentrates on the atomic-level control of the intrinsic electronic structure of carbon materials; both strategies have demonstrated significant performance improvements in experiments. Their experimental performance results are compared in Table 3.

**Table 3:** Comprehensive comparison of rare-earth-modified carbon electrodes: preparation methods, test conditions, performance metrics, and cycling stability.

Materials	Test Current Density	Core Test Indexes	Detailed Experimental Performance Results	Reference	Preparation Method	Cycling Stability
Pristine graphite felt	100/200 mA·cm^−2^	Energy efficiency, charge–discharge polarization, cycling stability	At 100 mA·cm^−2^, EE ≈ 73.3%; at 200 mA·cm^−2^, EE only 53.9%. Severe polarization at high current; energy efficiency drops sharply after long cycling due to fiber corrosion and loss of surface functional groups [32]	[32]	As-received commercial GF; no modification (baseline)	EE decays~15–20% after 100 cycles at 100 mA·cm^−2^; fiber corrosion observed
0.2 wt% CeO_2_ modified graphite felt	200 mA·cm^−2^	Energy efficiency, vanadium redox catalytic activity	EE reaches 64.7%, 10.8% higher than pristine GF; Ce^3+^/Ce^4+^ electron shuttle significantly accelerates VO^2+^/VO_2_^+^ reaction kinetics; optimal loading balance between active sites and mass transport [32]	[32]	Hydrothermal synthesis of CeO_2_ nanoparticles; impregnation-coating on GF; calcination at 400 °C in N_2_	Stable over 200 cycles at 200 mA·cm^−2^; EE retention > 92%; minor CeO_2_ particle detachment after 300 cycles
Gd-doped CeO_2_/GF composite electrode	160 mA·cm^−2^	Energy efficiency, catalytic synergistic effect	Energy efficiency increases by 4.5% compared with single CeO_2_ modified graphite felt; Gd doping introduces extra lattice defects and oxygen vacancies via charge compensation (Gd^3+^ → Ce^4+^) [24]	[24]	Hydrothermal co-precipitation of Ce_1−x_Gd_x_O_2_ nanoparticles; dip-coating on HT-GF; 500 °C calcination	Stable over 300 cycles at 160 mA·cm^−2^; EE retention >95%; Gd doping suppresses CeO_2_ phase segregation
Sm-doped CeO_2_/GF composite electrode	160 mA·cm^−2^	Energy efficiency, interfacial charge transfer	Energy efficiency rises by 7.8% relative to pure CeO_2_/GF; Sm optimizes carbon fiber interface electronic structure; smaller ionic radius of Sm^3+^ induces greater lattice distortion than Gd^3+^ [24]	[24]	Hydrothermal co-precipitation of Ce_1−x_Sm_x_O_2_ nanoparticles; dip-coating on HT-GF; 500 °C calcination	Stable over 300 cycles at 160 mA·cm^−2^; EE retention > 96%; Sm-doped solid solution shows excellent structural stability
Multi-rare-earth co-doped carbon electrode	100 mA·cm^−2^	Charge transfer resistance, CV peak current	Reduces charge transfer impedance by 65% compared with unmodified carbon felt; redox peak current reaches 3.96 × 10^−3^ A; synergistic effect of multiple rare-earth ions creates high-density active sites [33]	[33]	High-temperature heat treatment (>800 °C) with mixed rare-earth precursors; in situ lattice doping during carbonization	Stable over 500 cycles at 100 mA·cm^−2^; EE retention > 97%; bulk doping eliminates active site leaching
MnCo@C derived from bimetallic MOF	100 mA·cm^−2^	Energy efficiency, capacity retention, high-current tolerance	EE = 82.2%, 9% higher than raw carbon felt, 5% higher than single MnO@C/Co@C electrodes; after 500 cycles, capacity retention is 15% higher than pristine CF; MOF-derived porous carbon provides hierarchical structure [4]	[4]	Bimetallic Mn/Co MOF precursor synthesis; pyrolysis at 700–900 °C under Ar; in situ carbon encapsulation	500 cycles with capacity retention 15% higher than pristine CF; EE decay < 5% over 500 cycles
BFAC bifunctional activated carbon graphite felt	200/300 mA·cm^−2^	Energy efficiency, mass transfer performance	EE = 78.6% at 200 mA·cm^−2^; can stably operate at 300 mA·cm^−2^ with EE of 69.7%; hierarchical porous structure enhances electrolyte penetration and ion transport at high current density [1]	[1]	Biomass-derived activated carbon; KOH activation; coating on GF via vacuum infiltration	Stable at 300 mA·cm^−2^ for 200 cycles; EE retention > 90%; robust activated carbon coating
CeO_2_/carbon nanofiber composite electrode	50–200 mA·cm^−2^	Cathode redox catalytic performance	Greatly improves VO^2+^/VO_2_^+^ reaction activity due to substantial increase in electrochemical specific surface area; has limited promotion effect on V^2+^/V^3+^ anode reaction; CeO_2_ nanoparticles uniformly embedded in PAN-based carbon nanofibers [34]	[34]	Electrospinning of PAN/Ce precursor; stabilization at 280 °C; carbonization at 1000 °C in N_2_	Stable over 200 cycles; no significant CeO_2_ leaching due to embedded structure in carbon nanofibers
Bi/CeO_2_ heterojunction composite electrode	100/200 mA·cm^−2^	Dual-pole bifunctional catalysis	Bi acts as V^2+^/V^3+^ catalyst, CeO_2_ catalyzes positive VO^2+^/VO_2_^+^ reaction; heterojunction electron redistribution reduces overall cell polarization loss; achieves simultaneous optimization of both electrode reactions [35]	[35]	Solvothermal synthesis of Bi/CeO_2_ heterostructure; ultrasonic-assisted deposition on GF; 350 °C annealing	Stable over 400 cycles at 100 mA·cm^−2^; heterojunction interface prevents Bi dissolution; EE retention >94%
Rare-earth porous spinel Co_3_O_4_ electrode with atomic Ce doping	Industrial high current density	Overpotential, long-term stability	Ultra-low reaction overpotential under acidic electrolyte; continuous stable operation over 550 h; Ce atomic doping induces active site migration and enhances mass transfer; hierarchical porous spinel structure [36]	[36]	Template-assisted synthesis of porous Co_3_O_4_ spinel; atomic Ce doping via wet impregnation; 600 °C calcination	Continuous stable operation > 550 h at industrial current density; overpotential increase < 10 mV
La^3+^ bulk-doped carbon felt	100/200 mA·cm^−2^	Charge transfer resistance, wettability, cycling stability	Charge transfer resistance reduced by 42%; water contact angle decreased from 128° to 47°; La^3+^ lattice distortion creates abundant edge defects; stable operation over 300 cycles with <3% EE decay [25]	[25]	High-temperature diffusion doping of La(NO_3_)_3_ precursor into carbon felt; 900 °C heat treatment in Ar	Stable over 300 cycles with <3% EE decay; La^3+^ firmly anchored in carbon lattice; no detectable leaching
CeO_2_–Bi_2_O_3_ ternary composite on carbon cloth	150/250 mA·cm^−2^	Energy efficiency, high-current performance, capacity decay rate	EE = 80.1% at 150 mA·cm^−2^; maintains 68.3% at 250 mA·cm^−2^; ternary heterojunction achieves triple-functional catalysis; capacity decay rate reduced to 0.012% per cycle over 1000 cycles [35]	[35]	One-pot solvothermal synthesis of CeO_2_–Bi_2_O_3_ ternary composite; vacuum filtration on carbon cloth; 400 °C annealing	1000 cycles with capacity decay rate 0.012%/cycle; ternary heterojunction shows exceptional durability

### 3.1. Rare-Earth Oxide Modification Strategies

The modification of electrodes using rare-earth oxide nanoparticles is the most widely applied and technically mature method; amongst these, CeO_2_ has been the most extensively studied modification material due to its abundance of reversible oxygen vacancies and excellent Ce^3+^/Ce^4+^ redox cycling properties. This method utilizes techniques such as hydrothermal synthesis, precipitation–calcination, electrochemical deposition and impregnation-coating [18,19,37,38,39] to uniformly load rare-earth oxide nanoparticles onto the surfaces of carbon-based electrodes, such as graphite felt and carbon paper, thereby functionalizing the electrodes.

**Figure 2:**
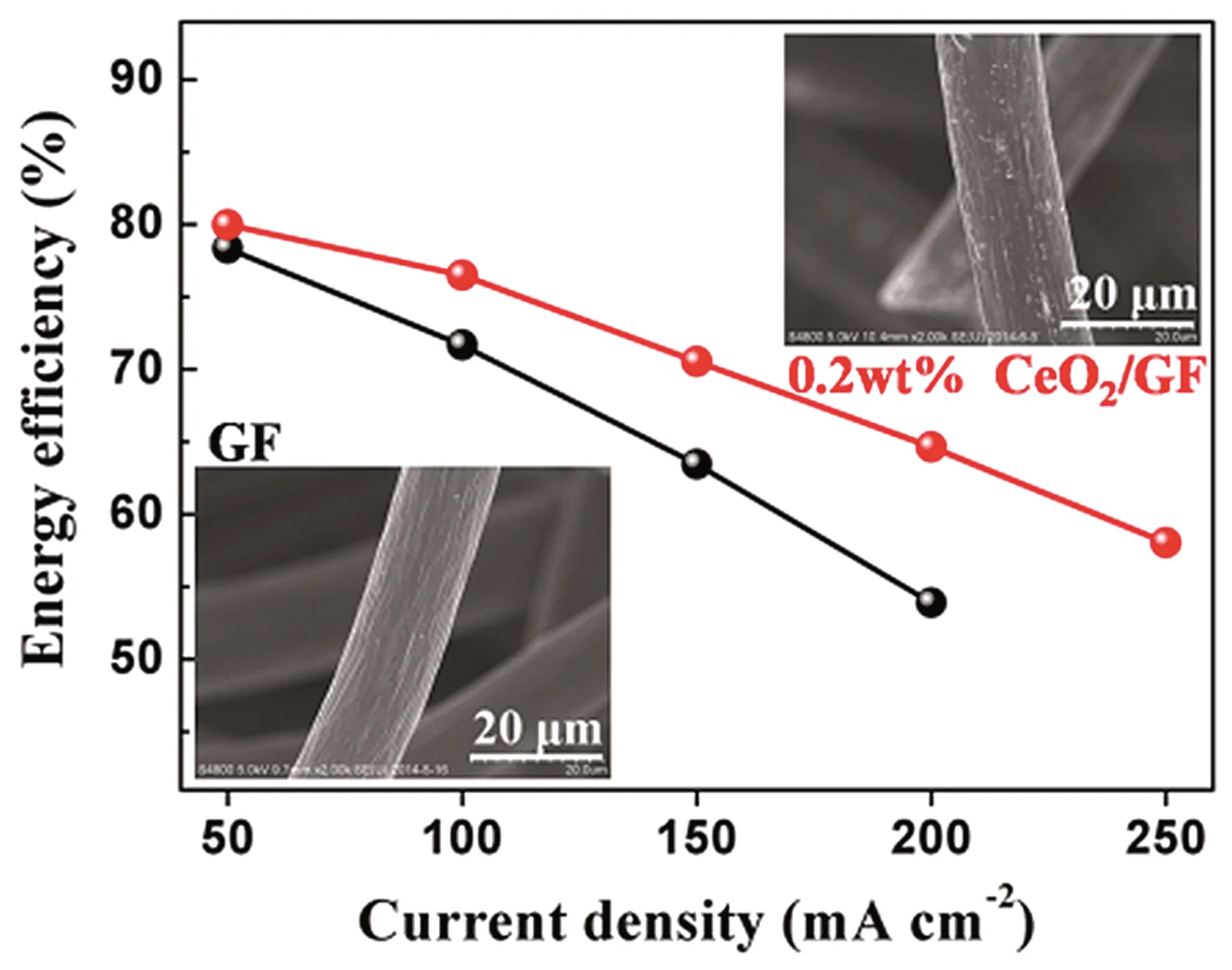
Energy density graph of cerium oxide-modified graphite electrode [32].

The oxygen vacancies on the surface of rare-earth oxides act as catalytic centers, adsorbing and activating vanadium oxygen ions; reversible electron pairs such as Ce^3+^/Ce^4+^ function as ‘electron shuttles’, establishing efficient charge transfer pathways, reducing the overpotential of the VO^2+^/VO_2_^+^ reaction, and accelerating interfacial electron transfer [16,40,41]. This strategy is highly effective, as demonstrated by Qiu et al. [32] (Table 3), where 0.2 wt% CeO_2_-modified graphite felt achieved an EE of 64.7% at 200 mA·cm^−2^, 10.8% higher than the pristine GF (53.9%). VRFB single-cell testing indicated that, at a current density of 200 mA·cm^−2^, the 0.2 wt% CeO_2_/GF sample exhibited the highest energy efficiency of 64.7%, significantly higher than that of the pristine GF (53.9%) in Figure 2.

In addition to CeO_2_, rare-earth oxides with multivalent properties, such as Pr/Tb (Table 2), have also been shown to enable efficient modification of carbon-based electrodes via similar mechanisms; among these, multivalent rare-earth elements can simultaneously exhibit excellent catalytic activity for vanadium ion reactions at both the anode and cathode [19].

### 3.2. Bulk Doping Strategies for Rare-Earth Elements

Unlike surface modification, which relies on external active sites on carbon materials, bulk doping with rare-earth elements shifts the focus of modification to the interior. Methods such as high-temperature heat treatment, hydrothermal synthesis or chemical vapor deposition are employed to introduce ions such as La^3+^, Gd^3+^ and Ce^3+^ into the carbon lattice or defect structures, thereby enabling atomic-level control of electronic states, ultimately reshaping and enhancing the catalytic activity of the carbon matrix at its very source [20,21,22,23,25]. This method, although more complex, eliminates the leaching of active sites common in surface modification, offering superior stability [42,43].

For validation, hydrothermal methods can prepare modified graphite felt electrodes with Gd- or Sm-doped CeO_2_ nanoparticles, demonstrating energy efficiency improvements of 4.5% and 7.8% (Table 3), respectively, at 160 mA cm^−2^ [24]. The full fabrication procedure of Ce_1-x_M_x_O_2_/GF via hydrothermal treatment is described below. Pristine GF was first calcined at 500 °C for 5 h under an air atmosphere to improve hydrophilicity, yielding heat-treated HT-GF. Water contact angle characterization confirms ultra-fast adsorption of water droplets on HT-GF surfaces, revealing substantially enhanced wettability relative to unmodified GF. HT-GF was then adopted as the substrate, and a functional modification layer was grown through hydrothermal reaction to obtain Ce_1-x_M_x_O_2_/GF. Microscopic morphology observations show that carbon fibers in the original GF possess smooth surfaces with limited defects. After thermal treatment, HT-GF displays remarkably rough fiber surfaces abundant in pores, which enlarge the electrochemically active area and benefit battery performance. For electrodes with varied dopants, both CeO_2_/GF and Ce_0.8_Sm_0.2_O_2_/GF preserve partial pores generated during high-temperature calcination. Nevertheless, Ce_0.8_Sm_0.2_O_2_/GF exhibits a relatively smoother surface with reduced pore quantity, indicative of loosely packed surface nanoparticles. Comparatively, CeO_2_/GF contains fewer pores than Ce_0.8_Sm_0.2_O_2_/GF, implying denser nanoparticle deposition on graphite felt fibers for the latter sample. Cross-sectional SEM characterization further verifies complete coverage of carbon fibers by Ce_0.8_Sm_0.2_O_2_ nanoparticles. Elemental characterization via EDS confirms the presence of C, O, Ce and Sm within Ce_0.8_Sm_0.2_O_2_/GF, alongside homogeneous element distribution over carbon fiber surfaces.

The benefits of doping are also shown by Li et al. [33] (Table 3), where co-doping with rare-earth elements significantly reduced charge transfer impedance by 65%, with peak redox current reaching 3.96 × 10^−3^ A, highlighting the strong validation through experimental characterization.

Rare-earth doping optimizes the energy level matching with the reaction orbitals of vanadium ions by regulating the distribution of the carbon framework’s electron cloud and the Fermi level, thereby reducing the interfacial charge transfer barrier; Large-radius rare-earth ions induce lattice distortion, leading to the generation of a large number of topological defects, edge active sites and pore structures, thereby achieving high-density construction of active sites; rare-earth ions form stable coordinate bonds with oxygen-containing functional groups on the carbon surface, suppressing the loss of functional groups and high-temperature graphitisation stacking, and maintaining the porous structure and long-term cycling stability [26]. Compared with surface modification, bulk doping enables atomic-level dispersion of rare-earth elements, eliminating particle agglomeration and the leaching of active sites and thus offering superior long-term stability. However, the preparation process for this method is complex, and precise control of the doping concentration is challenging; consequently, research remains largely confined to the laboratory, and scaling up for engineering applications still requires overcoming technical bottlenecks in the preparation process [42,43].

## 4. Synthesis and Catalytic Effects of Rare-Earth-Based Composite Catalysts

To further push beyond the performance limits of single-rare-earth modification strategies, researchers have developed a variety of rare-earth-based composite electrocatalysts through component blending and nanostructure design. By utilizing the synergistic effects between multiple components, they have simultaneously optimized catalytic activity, electronic conductivity and structural stability, thereby meeting the application requirements of vanadium flow batteries for high current density and long-cycle cycling. Specific experimental performance results of these composite systems (e.g., CeO_2_/carbon nanofibers, Bi/CeO_2_ heterojunctions) are detailed in Table 3. The most extensively studied composite systems to date are as follows:

### 4.1. Rare-Earth-Carbon Nanomaterial Composite Systems

This system combines rare-earth oxides, such as CeO_2_, with carbon nanomaterials including carbon nanotubes, reduced graphene oxide and carbon nanofibers. It fully integrates the excellent electrical conductivity and high specific surface area of carbon nanomaterials with the high catalytic activity of rare-earth materials, thereby achieving dual optimization of the conductive network and active sites [44]. As shown in Table 3, embedding CeO_2_ nanoparticles within polyacrylonitrile (PAN)-based carbon nanofibers (CeO_2_/ECNFs) improved the electrocatalytic activity, primarily attributed to the substantial increase in the electrochemical specific surface area [34]. Based on cyclic voltammetry (CV) and electrochemical impedance spectroscopy (EIS) results, the addition of CeO_2_ improved the electrocatalytic activity of the vanadium flow battery’s cathodic reaction to a certain extent, whilst having a minimal impact on the anodic reaction. The significant improvement in the electrochemical performance of CeO_2_/ECNFs is primarily attributed to the substantial increase in the electrochemical specific surface area.

### 4.2. Rare-Earth-Metal Heterostructure Catalysts

This system combines rare-earth oxides with non-precious metals such as Bi, Pb and Nb, or precious metals such as Pt and Ir, to form heterojunctions. These composite systems (e.g., Bi/CeO_2_ heterojunctions in Table 3) utilize electron redistribution to optimize vanadium ion redox kinetics at both electrodes [35]. By utilizing electron redistribution and strong electron interactions at the heterointerface, it simultaneously optimizes the kinetics of vanadium ion redox reactions at both the anode and cathode, thereby achieving bifunctional synergistic catalysis [45,46,47]. Among these, non-precious metal–rare-earth heterojunctions are currently a hot topic of research, offering the dual advantages of high performance and low cost. For example, in Bi/CeO_2_ heterojunction composites (Table 3), Bi nanoparticles catalyze the positive reaction, while CeO_2_ acts as the cathode catalyst, achieving bifunctional synergistic catalysis [35].

Among these, non-precious metal–rare-earth heterojunctions are currently a hot topic of research, offering the dual advantages of high performance and low cost. For example, in Bi/CeO_2_ heterojunction composites, Bi nanoparticles themselves exhibit excellent catalytic activity for the V^3+^/V^2+^ reaction at the cathode, whilst CeO_2_ demonstrates outstanding catalytic performance for the VO^2+^/VO_2_^+^ reaction at the anode; the charge transfer effect at the heterojunction interface further optimizes the electronic structure of both components, whilst enhancing the catalytic activity of the anode and cathode reactions, thereby achieving a significant reduction in the polarization loss of the entire cell [35].

Although precious metal–rare-earth composite systems exhibit superior catalytic activity, they are currently confined to basic laboratory research due to the high cost of precious metals, making large-scale application difficult to achieve.

### 4.3. Porous Rare-Earth-Based Catalytic Materials

Mesoporous/macroporous single or mixed rare-earth oxides prepared via the template method and self-assembly techniques possess a hierarchical porous structure and an ultra-high specific surface area. The benefits of such architectures are highlighted by the porous spinel Co_3_O_4_ electrode with atomic Ce doping (Table 3), which not only exhibited low overpotential but also demonstrated industrial-level stable operation for over 550 h [36]. This not only maximizes the exposure of catalytic active sites but also provides continuous, rapid pathways for electrolyte permeation and ion transport, making them particularly well-suited to the reaction requirements of vanadium flow batteries operating at high current densities [48,49,50]. The Ce induces the migration of active sites and enhances mass transfer; under acidic conditions, the overpotential is extremely low, and the catalyst operates stably for over 550 h at industrial current densities.

## 5. Analysis of the Mechanism of Rare-Earth-Modified Electrodes

**Figure 3:**
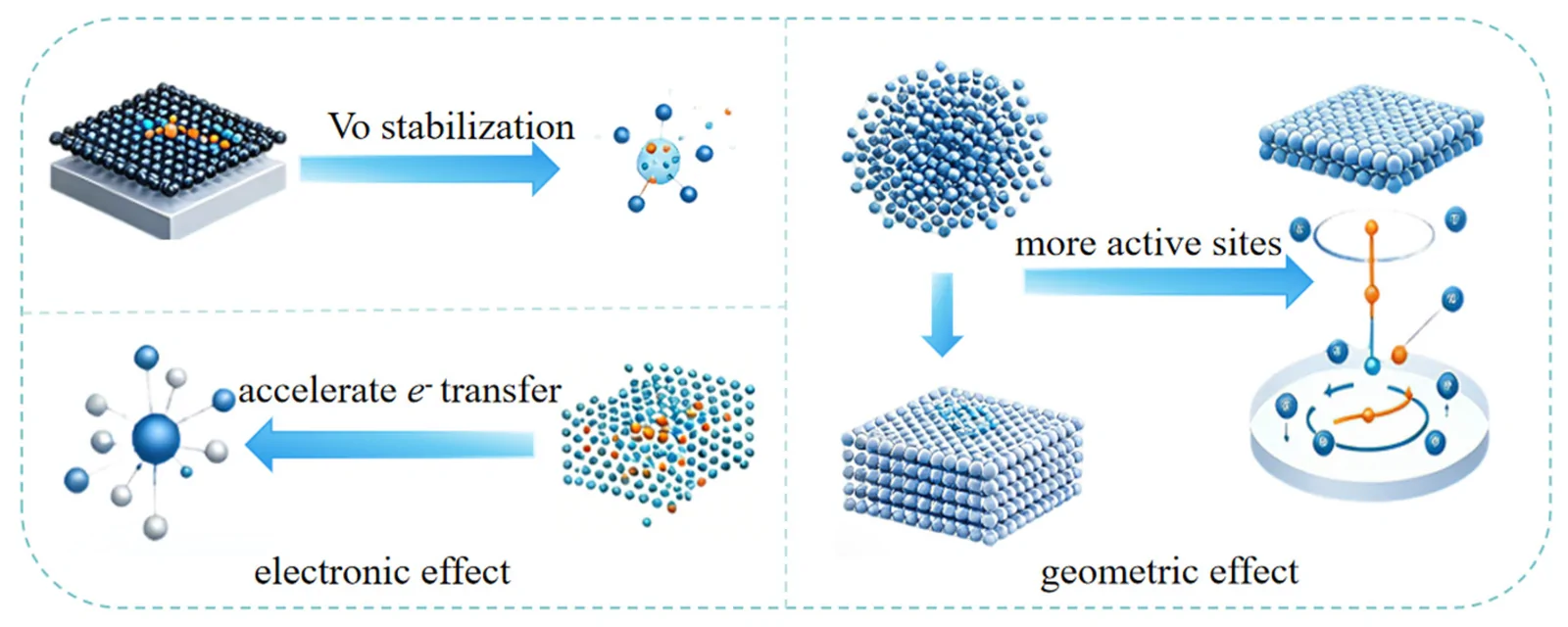
Reaction mechanism of a flow battery. The partial graphical elements were produced by AI image-generation tools. The authors have modified, reviewed and validated all scientific information contained in this figure to guarantee scientific accuracy.

The enhancement in the performance of vanadium flow battery electrodes achieved through the use of rare earths stems from the synergistic regulation of electronic and geometric effects [51,52,53]. This section improves upon theoretical aspects of how electronic phenomena, affected by geometric effects (Figure 3), are linked to rare-earth physicochemical properties, morphology, and performance (Table 3). The electronic effect reduces the activation energy of the vanadium ion redox reaction and accelerates interfacial charge transfer by modulating the work function and Fermi level of the carbon electrode, utilizing the ‘electron shuttle’ role of rare-earth variable-valence ions, and leveraging the stabilizing effect of oxygen vacancies on reaction intermediates; this is central to enhancing the intrinsic catalytic activity of the electrode; geometric effects, on the other hand, rely on rare-earth nanoparticles and porous structures to fully expose active sites, improve electrode wettability and improve ion mass transfer efficiency, providing a continuous structural foundation for rapid pathways (Figure 3) and thus reinforcing the electronic effects [54,55,56,57]. The coupled action of these two effects simultaneously optimizes the number of active sites, catalytic activity, electron conduction and ion transport capabilities, fundamentally resolving the key issues of sluggish reaction kinetics and insufficient long-term stability associated with carbon-based electrodes [58,59,60,61,62,63,64,65,66,67]. For instance, Figure 3 illustrates how rare-earth atoms drive active site relocation to enhance mass transfer and lower reaction overpotential in acidic media [36].

## 6. Challenges and Outlook

Significant progress has been made in the use of functionalized rare-earth materials for electrode modification in vanadium flow batteries; however, the transition to large-scale engineering applications still faces numerous key challenges. These include the as yet unclear mechanisms governing the long-term stability of rare-earth active centres under harsh operating conditions; the relatively high cost of high-purity rare earths and composite systems; the difficulty in adapting existing preparation processes to industrial-scale production; insufficient performance validation and lifespan assessment under full-cell and real-world operating conditions; and the fact that research into catalytic mechanisms largely relies on off-line characterization, lacking support from in situ dynamic evidence; Future research should focus on the rational design of highly stable, low-cost rare-earth-based catalytic systems; the development of in situ multi-scale characterization techniques; the optimization of scalable, continuous preparation processes; engineering validation at the full-cell and stack levels; and the establishment of a rare-earth full-lifecycle recycling system.

Specifically, in light of circular economy aspects, incorporating discussion on life servicing and recycling strategies is critical. Selected rare-earth treatment methods should be evaluated not only for initial performance, as seen in Table 3, but also for their long-term stability and compatibility with recycling protocols. Full-lifecycle recycling of rare-earth components from spent catalytic electrodes offers an innovative pathway towards environmental sustainability and economic feasibility for industrial-scale deployment, leveraging lifecycle recycling and reuse of rare earths to reduce total battery manufacturing costs.

## 7. Conclusions

Benefiting from their distinctive 4f electronic configuration, abundant surface defect sites and outstanding interfacial modulation performance, rare-earth-functionalized materials provide a transformative technical route to address the critical limitation of sluggish vanadium redox kinetics afflicting carbon electrodes of vanadium redox flow batteries (VRFBs). This review systematically classifies two distinct rare-earth modification approaches—surface loading and lattice doping—clarifying their differences in active site formation, electronic regulation logic, and cycling stability. By adopting multiple modification approaches, including surface nanocoating, bulk lattice doping and multicomponent composite construction, as detailed in Table 3, coupled with the synergistic modulation of electronic and geometric effects established in our comprehensive theoretical framework (Figure 3), the catalytic activity, electronic conductivity, ion mass transfer capacity and cycling durability of carbon electrodes can be comprehensively upgraded, rendering rare-earth modification a prominent research hotspot in VRFB electrode material domain.

As in-depth insights into catalytic mechanisms are gained, material design strategies are continuously optimized, and scalable manufacturing technologies achieve progressive breakthroughs, rare-earth-functionalized materials will power the advancement of VRFBs toward high power density, superior energy efficiency, ultra-long service life and low manufacturing cost. This work, by establishing a solid theoretical foundation and linkage between rare-earth physicochemical properties, morphology (Figure 3), and performance (Table 3), provides targeted guidance for rational design of high-performance rare-earth-modified electrodes. Innovatively proposing full-lifecycle recycling pathways (Section 6), such technological progress will further expedite the large-scale commercial deployment of VRFBs within long-duration grid energy storage, delivering solid material and technical backing to advance the low-carbon energy transition and accelerate the full implementation of China’s ‘Dual Carbon’ strategic objectives.

## Data Availability

No new data were created or analyzed in this study. Data sharing is not applicable to this article.
